# Solvent-Free Melting Techniques for the Preparation of Lipid-Based Solid Oral Formulations

**DOI:** 10.1007/s11095-015-1661-y

**Published:** 2015-03-19

**Authors:** Karin Becker, Sharareh Salar-Behzadi, Andreas Zimmer

**Affiliations:** 1 Institute of Pharmaceutical Sciences, Department of Pharmaceutical Technology, Karl-Franzens-University Graz, Member of BioTechMed, Universitätplatz 1, 8010 Graz, Austria; 2 Research Center Pharmaceutical Engineering GmbH, Inffeldgasse 13, 8010 Graz, Austria

**Keywords:** agglomeration, coating, extrusion, lipid, melt, solvent-free

## Abstract

Lipid excipients are applied for numerous purposes such as taste masking, controlled release, improvement of swallowability and moisture protection. Several melting techniques have evolved in the last decades. Common examples are melt coating, melt granulation and melt extrusion. The required equipment ranges from ordinary glass beakers for lab scale up to large machines such as fluid bed coaters, spray dryers or extruders. This allows for upscaling to pilot or production scale. Solvent free melt processing provides a cost-effective, time-saving and eco-friendly method for the food and pharmaceutical industries. This review intends to give a critical overview of the published literature on experiences, formulations and challenges and to show possibilities for future developments in this promising field. Moreover, it should serve as a guide for selecting the best excipients and manufacturing techniques for the development of a product with specific properties using solvent free melt processing.

## Introduction

The importance of lipid-based solid oral formulations has increased during the last decades, due to their outstanding benefits such as providing modified release profiles or taste masking using solvent-free processing techniques. Lipid-based excipients were first used in the 1960s for embedding drugs in a wax matrix in order to sustain drug release (1,2). In the more recent years these excipients were successfully used in oral drug delivery systems to enhance the bioavailability of poorly aqueous soluble drugs (3–5). Furthermore, taste masking and the improvement of swallowability have been achieved with these excipients (6). Further reasons for the application of lipids in a formulation may be (I) shelf life extension by protecting the drug from other ingredients or from environmental influences (II) the reduction of gastric irritation (III) the improvement of general attributes like flowability, lubrication performance, compressibility or mechanical resistance (7,8). Common techniques to obtain solid lipid-based formulations are: spray congealing/drying, adsorption on solid carriers, melt granulation/melt extrusion, supercritical fluid based methods, or processing of solid lipid nanoparticles (9). The major difference between these methods is whether or not solvents are applied during the process. Several formulations described in the literature are prepared by spraying techniques involving lipid excipients emulsified in water or dissolved in organic solvents and also aqueous dispersions of solid lipid nanoparticles (9–12). However, there are several drawbacks associated with the presence of solvents. An aqueous medium requires a time-consuming solvent evaporation step and residual moisture may affect product stability due to drug hydrolysis. Organic solvents offer a considerably faster evaporation step, though these solvents are much more expensive, flammable and often toxic. This causes other issues like the prevention of hazards to employees and environment, and additional costly steps for solvent recovery and disposal (13). On the contrary, working with molten excipients provides the outstanding benefit of evading any solvent. Thereby, all the described disadvantages can be overcome. Despite the promising benefits, there are also some problems involved with the application of solvent-free melting techniques with lipid based excipients, *e.g.,* heat sensitive drugs may undergo degradation and polymorphic and morphological changes of the lipid or crystallinity changes of the drug may alter the product performance over time.

Countless studies on the development of solid oral formulations using solvent-free melting techniques have been published in the last decades. The current work is a critical review of these studies based on the applied excipients, processing strategies and the analytical methods for the characterization of the excipients and the end product. The focus is on manufacturing processes, which are categorized into three main strategies, *i.e.,* melt extrusion, melt coating and melt agglomeration. Additionally, this review intends to give support for selecting appropriate lipid excipients and suitable solvent-free manufacturing techniques to obtain the desired end product.

## Lipid-Based Excipients

Substances containing a fatty acid in their chemical structure are referred to as lipid-based excipients. Even though fatty acids occur naturally and are part of the daily diet, a general statement about the safety and toxicity of their derivatives cannot be made. A reasonable approach is the selection of lipid-based excipients listed as “generally recognized as safe” (GRAS) or registered as inactive ingredient in the “inactive ingredient guide (IIG)” published by the United States Food and Drug Administration (FDA) or the use of food excipients, which only require a simplified authorization procedure for pharmaceutical application (14–16). However, physiological and biopharmaceutical interactions with other formulation compounds as well as additional toxicological effects of the formulation are not taken into account (14,17). Many lipophilic compounds act as substrates for the P-glycoprotein (PgP), a membrane-bound ATP-dependent efflux pump in the enterocyte that transports xenobiotics back into the intestinal lumen, and for the intestinal cytochrome P450 enzyme, a hydroxylase that metabolizes xenobiotics to more water soluble, often inactive derivatives that can be excreted through the urinary bladder (18,19). Their inhibition increases the absorption of drugs out of the intestinal lumen and reduces the metabolization into inactive drug derivatives leading to an overall higher drug exposure, which may go along with more pronounced side effects (18). An *in vitro* study, for instance, revealed that lipid-based additives such as Peceol and Gelucire 44/14, both approved as GRAS, inhibit the PgP-mediated efflux in human colon adenocarcinoma cells (Caco-2) by decreasing the PgP-protein expression (20). However, the prediction of the real *in vivo* impact is hardly possible, as it strongly depends on the applied excipient concentration, the binding-affinity, the therapeutic index of the drug, and even on the individual genetic protein polymorphism (18,21).

Nevertheless, the number of possible combinations of the molecular structure is virtually infinite, depending on the fatty acid (*e.g.,* chain length, the grade of saturation, the presence of branches, the kind of chemical bond (*e.g.,* ether/ester) and nature and number of the linked molecule (*e.g.,* glycerol, additional fatty acids, propylene glycol, polyglycerol, sucrose *etc.*). This leads to a wide field of application possibilities of these excipients comprising solubility enhancement as well as controlled drug release. A useful indicator to estimate the drug release is the hydrophilic-lipophilic balance (HLB) a numerical rating scheme based on the water solubility and polarity of the excipient (22,23). As the contact angle and, consequently, the wetting behavior are affected by the HLB, excipients with a lower HLB (≤5) can be expected to retard the original drug dissolution pattern (24). Apart from polarity the gel-formation properties, melting point, crystallinity and porosity as well as the nature of the drug (*e.g.,* solubility, melting point) itself and the way the drug is integrated into the formulation (*e.g.,* core/shell, matrix), can play an integral role in *in vitro* drug release (24–26). Table I gives an overview of the substance classes and associated materials applied as excipients in published formulations for both pharmaceutical and food supplementary products. The most frequently, purely used excipients in the recent past are Compritol 888 ATO for controlled release, Precirol ATO 5 (depending on coating amount and drug (27,28)) for taste masking, Gelucire 50/13 for solubility enhancement of poor soluble drugs, and stearic acid for pH-dependent release (*e.g.,* enteric coating). In particular, Precirol ATO 5 and Gelucire 50/13 are prone to exhibit polymorphic changes and have therefore been associated with storage instabilities. “Strategies for controlling polymorphism and formulation stability” lists a few strategies to circumvent this issue.

**Table I:** Overview of Lipid Based Excipients and Their Application in Literature

Substance class	Materials and application examples in literature
Waxes	Bees wax (29–34), carnauba wax (29,31,32,35–41)
Fully or partially hydrogenated vegetable oils and fats	Hydrogenated coco-glycerides (*e.g.,* Witocan 42/44, Witepsol E 85) (42–45), hydrogenated palm fat and oil (*e.g.,* Softisan 154) (46,47), hydrogenated castor oil (*e.g.,* Cutina HR) (48–58), hydrogenated rape oil (59), hydrogenated cottonseed oil (*e.g.,* Sterotex, Lubritab) (60–62), hydrogenated soybean oil (*e.g.,* Sterotex HM) (63–67), hardened soybean oil (*e.g.,* Dynasan 120) (68)
Polyoxylglycerides	Gelucire 55/18 (69,70), Gelucire 50/13 (71–85), Gelucire 44/14 (75,86–101), Gelucire 39/01 (102–104), Gelucire 43/01 (104–107), Gelucire 50/02 (28, 69,88) and Gelucire 64/02 (108), Compritol HD 5 (109)
Fatty acids	Myristic acid (59), palmitic acid (110–113), stearic acid (35,36,57–59,111,113–119), behenic acid (59, 120), Syncrowax AW1-C, Emulsifying wax NF (37)
Monoacylglycerides	Glyceryl monostearate (*e.g.,* Imwitor 491, Atmul 84 K) (39,55, 57,114, 115,121–132), glyceryl monooleate (133,134), glycerol monolaurate (84)
Diacylglycerides	Glyceryl palmitostearate (Precirol ATO 5) (27, 43,135–142) and glyceryl dibehenate (Compritol 888 ATO) (29,137,44,54,139,142–158) with 40–60% of diacylglycerides
Triacylglycerides	Trilaurin (Dynasan 112) (159,160), trimyristin (Dynasan 114) (44,141,142), tripalmitin (Dynasan 116) (159–162), tristearin (Dynasan 118) (45,131, 159,163–165), tribehenin (*e.g.,* Syncrowax HR-C), triglyceride of long fatty acids (Syncrowax HGL-C) (37,147)
Animal fats	Cow ghee (41,166,167)
Polyglycerides	Tetraglycerol pentastearate, tetraglycerol monostearate (168), polyglyceryl-6-stearate (47)
PEG fatty acid esters	PEG-6-stearate (*e.g.,* superpolystate) (169)
Sucrose fatty acid esters	Sucrose laurate (170,171), sucrose stearate (61,172), sucrose palmitate and sucrose behenate (173)

Common surfactants comprising a fatty acid in their molecular structure such as polyoxyethylene sorbitan fatty acid esters (Tween), sorbitan fatty acid esters (Span) or glyceryl esters of organic acids (E472 a-c) are excluded from the list. Waxy materials such as microcrystalline wax (hydrocarbon wax), carbowax and all other available polyethylene glycols are also excluded, as these materials do not comprise lipid-based structures. Nevertheless, these materials have been frequently processed with the within described melting techniques.

## Characterization Methods, Challenges and Controlling Strategies

Lipid-based excipients often involve acylglycerols in their structure or their blend. Acylglycerols exist in up to four different crystalline structures: the pseudo-hexagonal sub α-, the hexagonal α-, the orthorhombic β′- and the triclinic β-form (in order of increasing thermodynamic stability) (174–177). These forms not only differ in their packing density and stability, but also in their physico-chemical properties such as their melting point, recrystallization rate, and solubility in water (177–179). For instance, the transformation from the thermodynamically instable and less dense α-form to the most stable and densely packed β-form should result in the reduction of wettability. Another effect associated with polymorphic transformation occurs during the transformation from the hexagonal α- to the triclinic β-form while passing the orthorhombic β′-form. The altered geometric integrity of the crystalline structure leads to the spontaneous formation of flake-like fractal structures on the surface also known as “blooming” (164). Interestingly, the impact of this alteration on drug release described in the literature points towards two different directions, both causing a change in drug release after storage and indicating formulation instability. On the one hand, the rough surface was described as super water-repellent with contact angles larger than 150°, meaning a significant reduction of wettability (180,181). Several studies have proven the formation of fractal structures on the surface of different lipid formulations with SEM images, which seems to be associated with an undesired retarded drug release after storage.  It is of particular interest, that all described formulation comprised mono-, di- or triacylglycerides in a extruded mixture with the drug (159,182). This effect was even able to overrule the dissolution enhancing effect of some surfactants (*e.g.,* glyceryl monostearate, HLB 3.8) (131). On the other hand, several formulations have been reported showing a significant increase in their dissolution rate after storage (80,102,183).

Interestingly, these formulations all included a compound with a high HLB and a lower melting point (*e.g.,* Caprol PGE 860), which might form an amorphous partition in the surrounding of the crystallized lipid structures. The storage above the melting point increases the mobility of this amorphous phase and induces phase separation and may result in an increased wettability. This effect can be promoted additionally by changes in the structure of the crystalline phase due to polymorphic transition during storage (“thermally induced phase separation-recrystallization”). These phenomena are highly dependent on the characteristics of the raw material (*e.g.,* melting point, miscibility, crystal structure) as well as on the temperature and mobility of the molecules. In particular Gelucire 50/13, which is a mixture of mono-, di- and triacylglycerides (C_12_-C_18_) and polyethylene glycol esters, is already known to show an increased release rate due to thermally induced phase separation and polymorphic transformation (184). The last example, Precirol ATO5, will emphasize, how complex and severe the impact of the polymorphic behavior on formulation stability can be. Hamdani *et al.* reported a complete loss of the prolonged release properties after storage at accelerated conditions of melt-granulated pellets comprising Phenylephrine, Lactose 450, Precirol ATO 5 and different amounts of unmelted Compritol 888 ATO. However, the storage at 25°C/60% r.h. decreased the dissolution rate significantly (138). Reitz *et al.* reported a similar impact on drug dissolution rate by using Precirol ATO 5 for manufacturing theophylline extrudates. Although the dissolution rate decreased in the first week, after 9 months it was significantly higher than directly after manufacturing (142). The mechanism behind these observations is not explained, but it is nonetheless possible that the formation of more stable polymorphs and fractal structures reduces the dissolution rate after storing at room temperature. The increase of dissolution rate observed after storage at higher temperatures lacks an explanation and has to be associated with the heterogeneous composition and sensibility to thermal treatment (141). Nevertheless, the formulation design (*e.g.,* melting technique, excipient composition, drug properties and interaction with excipients *etc.*) may also affect the drug release during storage.

As the dissolution profile is influenced by various effects and complex interactions, it is not surprising that there also exist stable formulations with excipients that caused storage instabilities in other studies (139,185). Hence, it is crucial to understand the physicochemical behavior of the raw materials and the interaction and impact on the properties of the processed formulation. Therefore, the following subchapters will focus on the characterization methods for the excipients and the product after thermal processing. Subchapter 3.3 intends to show how storage instabilities may occur and how they can already be prevented in the preformulation stage.

### Characterization of the Excipients

Changes in polymorphism and morphological structure as well as phase separation phenomena are probably the most common effects inducing formulation instabilities during storage. Therefore it is crucial to develop a deep understanding of the physicochemical properties of the selected excipients before and after melting, under different conditions (*e.g.,* time, temperature), and in mixtures with each other and with and without the drug. This step saves time and costs in the process development phase. Table II summarizes methods used to gain insight into physico-chemical properties.

**Table II:** Characterization Methods for Lipid Excipients

Method	Information	Application in development
DSC (also coupled with XRD or TGA)	Melting and recrystallization point/range, peak broadness, polymorphism, thermal behavior, melting/recrystallizing fractions, chemical stability	Selection of process temperatures, prediction of storage stability and process performance (agglomeration/ film formation)
Isothermal microcalorimetry	Monitoring of thermal events	Detection of polymorphic changes during storage
XRD	Crystallinity, morphology, polymorphism	Prediction of storage stability
Hot stage polarization microscopy (HSM)	Crystal growth under well-defined conditions, polymorphic transformation	Selection of cooling rate, coating quality, prediction of storage stability
FT-IR/ NIR/ Raman	Polymorphism (finger print), chemical composition	Product quality, process monitoring (PAT)
Goniometry contact angle	Wettability, surface tension	Solubility, dissolution rate
Rheometer	Melt viscosity (shear rate, temperature), thermoplastic behavior of excipients	Processability (spraying techniques), selection of process parameters
Texture analyzer	Brittleness, film adhesion, swelling behavior, conductivity,	Coating quality, stability
Dilatometry	Thermal expansion/contraction	Coating quality
Profilometry	Surface roughness, topographic analysis	Mouth feel
Penetrometry	Material strength, hardness	Mouth feel
Dynamic vapor sorption (DVS)	Water sorption/desorption	Moisture protection
Karl Fischer titrimetry	Water content	Moisture protection
Acid/Base titration	Saponification value Acid value	Calculation of HLB (186)
TD-NMR	Solid fat content (SFC)	Spreadability, firmness, mouth feel, processability and stability

### Characterization of the Product After Melt Processing

Apart from the well-established powder and surface-morphology characterization techniques (72,109,110,126,187,188), analytical *in vitro* tools are also available for the estimation of the subsequent *in vivo* dissolution behavior of the formulation in the body. Common *in vitro* methods for this purpose are dissolution tests with biorelevant media and lipolysis studies with pancreatin (182,189–193). Biorelevant media contains physiologically relevant surfactants including a mixture of bile salts (*e.g.,* sodium taurocholate), phospholipids, pancreatic lipase to simulate lipolysis, and buffer and salts are added to adjust the pH and the osmolarity of the medium to imitate physiological conditions (182). Witzleb *et al.* showed that the release from lipid-based matrices in biorelevant media highly depends on the structure of the lipid. Cetyl palmitate and glyceryl monostearate, for instance, exhibited a significantly faster release in biorelevant media than in HCl due to a different solubilization with surfactants and enzymatic degradation in case of glyceryl monostearate. Other lipid matrices such as glyceryl tripalmitate and glyceryl dibehenate indicated only a minor effect of the changed medium on drug release (182). In addition, lipolysis measurements revealed that lipid digestion of the pancreatic lipase does not only depend on the chain length, but also on the grade of esterification, on the solid state, and the ability for solubilization (182,194,195). Hence, it cannot be excluded that changes in crystallinity and solubility due to polymorphic transition may have a significant impact on *in vitro* and *in vivo* drug release profiles (182). Along with an optimal dissolution rate, taste masking is often an important objective in formulation development (33,43,196,197). This is usually evaluated in sensory studies with trained volunteers (33,198–200). Some studies also involve cats, which are sensitive to bitterness and scorn bitter food with insufficient taste masking (197). A simple *in vitro* method is the use of a disintegration tester (1 min stirring, pH 7,4) for the evaluation of the short time release amount in a pH close to that in the mouth (196), but also the results of a test of the first minutes of dissolution have been utilized (201). Another method reported is the mini column method, which represents a release test that attempts to simulate the anatomy and physiology of the oral cavity (201,202). A more sophisticated method is the measurement of dissolved samples with an electronic tongue, which consists of different potentiometric sensors and a pattern recognition system (203,204) (Table III).

**Table III:** Characterization methods for product after melt processing

Parameter	Methods
Size distribution, sphericity, shape	Sieve analysis, digital imaging, laser diffraction (76,188,198)
Friability	Friabilator (33,48)
Flowability, compactibility, compressibility	FT4 powder rheometer, flow time (funnel), angle of repose, graduated cylinder (tapped/ bulk density, hausner ratio, carr index), texture analyzer (tensile strength profile) (48,169,198)
Hardness	Hardness tester, texture analyzer (32, 48,68)
Porosity	Mercury porosimeter (142,188,302)
True density, specific surface area (BET)	Helium pycnometer (81,158,188)
Surface topography and morphology	Scanning electron microscopy (SEM) (179,188)
Microstructure	(Cryogenic) Transmission electron microscopy (TEM) (206) Synchrotron radiation computed microtomography (SRμ-CT) (157)
Surface structure	Atomic force microscopy (AFM), stereomicroscopy (179)
Coating thickness	Optical coherence tomography (OCT) Terahertz spectroscopy (265,267)
Surface polymorphism	Attenuated total reflection fourier transform infrared spectroscopy (ATR-FTIR) (44)
Elemental composition & homogeneity	Energy dispersive x-ray microanalysis EDX) (84,306)
Chemical distribution & homogeneity	Raman confocal microscopy (raman mapping) (126)
Atomic composition & homogeneity	X-ray photoelectron spectroscopy (XPS) (188,366)
Buoyancy lag time	Floatability (visualization/counting method) (83,106,133,148,149)
Non-invasive, *in vivo* floating behavior	γ-scintigraphy with radiolabeled technetium (^99m^Tc), radiographic incorporation studies (107)
Taste (*e.g.,* bitterness)	Sensory studies with trained or untrained volunteer collective, electronic tongue, short-time dissolution profiles by using disintegration tester (33,196,199,200,203,238)
Dissolution	Dissolution tester I/II, data fitting to mathematical kinetic models for controlled release (*e.g.,* zero order) or immediate release (30,57,106,188)
*In vitro* prediction of lipid digestion *in vivo*	Dissolution with biorelevant medium; *in vitro* lipolysis with pancreatin, construction of IVIVC-model in case *in vivo* data is available (9,182,189,190,191)
Storage stability at accelerated or intermediate conditions	Evaluation of quality attributes (*e.g.,* dissolution, taste) (43,91,138,139)

### Strategies for Controlling Polymorphism and Formulation Stability

Several approaches to control lipid polymorphism are conceivable, whereby only some have been applied in the existing literature:

- Tempering during processing:
  Process temperatures are kept between the melting point of the instable α- and stable β-form, in the area where the direct recrystallization of the stable β-form predominates over the β′-form (205). This prevents the formation of fractal structures (159) and the alteration of the dissolution profile due to morphological changes during storage. The recrystallization of the thermodynamically stable β-form is significantly slower than of the α-form. This may result in processing difficulties such as agglomeration in a fluid bed melt coating process or incomplete resolidification after spray chilling in a spray dryer. Melt extrusion is an appropriate technique to generate storage stable extrudates with this technique (142).
- Tempering after processing (“maturing”):
  In the first step recrystallization of the instable α-form is enabled by ensuring optimum process conditions. Maturing is performed after the process at elevated temperatures below but close to the melting point of the instable α-form. The maturing step is time consuming and may require additional equipment (*e.g.,* a drying chamber). Alternatively, a fluid bed coater can be used for that step, as this prevents the formation of agglomerates. The α → β transformation causes the formation of fractal structures and the alteration of the dissolution profile before and after maturing (64,66,146). A complete transformation is essential for achieving a storage stable formulation.
- Addition of crystallization seeds (“template effect”)
  The stable β-form or material with a comparable saturation grade and a chain length difference of *n* ≤ 4 can be used as seed material (177). The seed material is added to the molten lipid in the solid state. The presence of seeds acting as templates should accelerate the direct recrystallization of the molten material into the stable β-form (67,177). In case the stable β-form recrystallizes directly from the melt during the process, fractal structures should be avoided (159,164). When seed material is applied in form of a spraying suspension, the amount is limited to prevent nozzle clogging. The efficiency of this measure in accelerating the recrystallization and its effect on the purity of the stable phase haven’t been analyzed in detail yet.
- Avoidance of melting:
  The polymorphic transformation of most lipid excipients is monotropic. That means that if melting of the raw material, which is usually provided by the supplier in the stable β-form, can be avoided, the storage instabilities due to polymorphic changes can be eliminated. The challenge herein lies in the processability. Solid lipid excipients with a critical polymorphic behavior should either have a suitable viscosity or have to be combined with an excipient that has an appropriate viscosity and/or shows no polymorphism or transforms into the stable form during or immediately after resolidification (161,207–209). In the case of aiming at a controlled release formulation, the formation of a physical mixture between drug and lipid excipient might be the wrong path as the dissolution rate of the physical mixtures may be higher than that of the solidified melt-dispersion (140,210).
- Addition of polymorphic modifiers:
  Several emulsifiers are known to have a significant impact on the crystallization (*e.g.,* nucleation rate, crystal growth and morphology) and polymorphic transition of lipid excipients (211,212). Although numerous publications exist on this topic, the majority of these studies discuss applying commercially available additives, which often consist of a chemical mixture with high heterogeneity and differ in their composition between different suppliers and even batches (211). Additional effects such as their concentration, kind of lipid, degree of undercooling as well as the use of agitation may have a significant influence on the crystallization and polymorphic process (211). Most of the application examples derive from and are addressed to the food industry and only a few studies are specific to the pharmaceutical industry (131,162,213,214). There are several examples how modifiers can impact the polymorphism: Sucrose esters obstruct the α → β and β′ → β transition in tristearin and hydrogenate sunflower oil due to their high rigidity and some representatives (*e.g.,* P-170, S-170) also affect the crystal size of the high-melting fraction of milk fat (215–217). Garti *et al.* claimed that solid emulsifiers such as Span 40 (sorbitan monopalmitate), Span 60 (sorbitan monostearate), Span 65 (sorbitan tristearate) as well as glyceryl monostearate were able to stabilize the α-form and prevented the transformation into the stable β-form in tristearin (218,219). However, ageing experiments at room temperature revealed that only a few molecules (*e.g.,* triglycerol-l-stearate, sorbitan monostearate) with a suitable dimension of the hydrophilic moiety (“button syndrome”) were able to preserve a certain amount of the α-form for a longer storage time (218). As the effect on polymorphism depends on the chemical and structural nature of the lipid and emulsifier, in principle it is conceivable that the stabilization of the α-form during storage may be feasible to some extent. Nevertheless, this approach is associated with a high risk of transformation into the stable β-form in particular at higher storage temperatures. A more promising approach is the addition of emulsifiers to accelerate the transformation into the stable β-form. In particular, liquid or semi-solid emulsifiers such as different polysorbates (Tween 60, 65, 80) and sorbitan monolaurate (Span 20) were proven to promote the α → β transformation due to an increase in molecular mobility (218,220). In the pharmaceutical industry this approach was used to coat N-acetylcysteine in a fluid bed coater with a coating consisting of tripalmitin and polysorbate 65 (162). As an advantage, the process temperatures can be kept to a minimum, which is preferable for drugs sensitive to heat, and the transformation and morphological changes will be complete before storage. However, it must be borne in mind that phenomena apart from polymorphism can lead to storage instabilities in particular if a liquid or semi-solid emulsifier is used in a greater amount (*e.g.,* phase separation). Furthermore, the application of a higher amount of low-melting excipients can pose a problem if melt coating (*e.g.,* fluid bed coating) or fast recrystallization in general is required. Thus, it is wise to take the time and effort for pre-formulation studies exploring the polymorphic and morphological behavior (*e.g.,* crystal size, blooming, phase separation *etc.*) at different conditions (*e.g.,* temperature, cooling and recrystallization rates, concentration *etc.*).
- Selection of excipients without/with stable polymorphism
  Waxes such as carnauba wax, bees wax and stearyl stearate are stated to be non-polymorphic materials (40,46,221), which avoid storage instabilities associated with polymorphic changes. In particular the high melting point and brittleness of carnauba wax can induce processing problems such as nozzle clogging. Carnauba wax and bees wax have been most frequently used for controlled release formulations, but also immediate release is feasible with the addition of dissolution enhancers. Polyglycerides are said to be non-polymorphic, but stable in the α-crystalline form (222,223). The amount of glycerol- and esterified fatty acid molecules contributes to the HLB and can be adjusted in a wide polarity range and oligoglycerols with a degree of polymerization of up to 10 are approved by the FDA (224). Polyglycerol bears more functional groups for modification than PEG, and therefore shows a higher adaptability to different requirements such as the melting point or viscosity. However, these materials have been overlooked by the pharmaceutical industry for a long time (224).
- Selection of polymorphic excipients:
  Lipid excipients consisting of a complex mixture of glycerol molecules with different degrees of esterification (*e.g.,* Precirol ATO 5, Compritol 888 ATO, Gelucire 50/13 *etc.*) and/or of esters of different fatty acids (*e.g.,* Precirol ATO 5) should be treated with particular caution (70,225). The high heterogeneity leads to a complex polymorphic behavior difficult to predict during melt processing and storage (137). The polymorphic behavior of Compritol 888 ATO, which mainly consists of glyceryl dibehenate, has been studied extensively with DSC, time-resolved synchrotron x-ray diffraction and infrared spectroscopy at different conditions (*e.g.,* cooling rate, addition of pure acyl glycerides *etc.*) (135,226). These studies revealed that the ratio of mono-, di- and tribehenin and especially the monobehenin amount is important for the formation of a pseudohexagonal sub-α and hexagonal α-phase (226). In particular the less stable and compact sub-α-form shows a higher drug incorporation, which is desired in the preparation of solid lipid microparticles (226,227). Therefore a variation in the batch composition of the supplier as well as the mixture with excipients containing monoacylglycerides themselves can lead to changes in the equilibrium and different polymorphic behavior (226). Nevertheless, Compritol 888 ATO has been widely used in controlled release formulations and seems to be able to provide a stable release profile during storage (43,228). In contrast, the literature seems to lack polymorphic studies for Precirol ATO 5 with a similar degree of detail. Hamdani *et al.* mainly used DSC and powder X-ray diffraction to investigate the sensitivity of the polymorphism on thermal treatment of Precirol ATO 5 (137). The complex polymorphic behavior due to the high inhomogeneity of the mixture led to several studies that showed an altered dissolution profile (138,142). However, the higher complexity of the aforementioned materials also brings advantages such as the broader melting and recrystallization range, which leads to a better spreading and higher robustness against process changes compared to pure materials with a sharp recrystallization profile such as triacylglycerides (*e.g.,* Dynasan 114) (142).
- Specification of storage temperature:
  Transformation kinetics and phase separation phenomena are both temperature dependent (26,82). The selection and specification of adequate storage conditions might be the last way to prevent or at least slow them down to a minimum. However, this approach is less favored by the pharmaceutical industry as patient compliance is an issue.

## Selection of Lipid-Excipients and Melt Processing Techniques

Several techniques have been described in the literature on solvent-free preparation of lipid-based solid, oral formulations. Nevertheless, the first step in formulation development should always be a careful selection of the lipid excipients (and other excipients if necessary). A useful, rationale based approach is provided by the FDA in the ICH guideline Q8 within the framework of the concept of quality by design (QbD) (229). According to this guideline a quality target product profile (QTPP) should be defined for the final or intermediate product. This QTPP should include all desired characteristics such as the dosage form, drug load, particle size, bioavailability, dissolution behavior, taste and stability (230). Based on the QTPP the next step will be the definition of the critical quality attributes (CQA) for the excipients and the drug and their appropriate specification limits, which must be met to ensure the desired product quality and patient safety (229). Typical CQAs are, for instance, the dissolution rate, the particle size distribution, the maximum permissible impurity profile (231) as well as the stability of the formulation (232). Rosiaux *et al.* (26) gave an overview of formulation parameters (*e.g.,* HLB, excipient characteristics, further additives) that can be used to adjust the drug release from lipid matrices. Pore formers or surfactants with higher polarity accelerate the drug release, whereas increasing the concentration and melting point of the hydrophobic lipid excipient can be used to slow down the drug release (26). Taste masking with an immediate release profile can be achieved by applying lipids with a comparably low melting point such as Dynasan 114, Dynasan 116, Precirol ATO 5 or Witocan 42/44 (16,42,162,233–235). However, excipients with a melting point near room temperature may result in an inferior product quality due to poor flowability, a tendency to form great agglomerates and the risk of morphology changes during storage (236). Hence, comprising a liquid or a very low melting (<40°C) excipient in the formulation has to be well considered and the amount kept as low as possible. The ideal compromise between sufficient taste masking and immediate release may be found by applying a statistical design (DOE) to achieve an optimal adjustment of the critical process input parameters (CPP). Further consideration should be given to physical or chemical interactions between the chosen lipid excipients and the drug with special regard to temperature induced degradation and changes in crystallinity (solid solution, polymorphic changes). The selection of equipment and processing technique depends on the desired dosage form and particle size distribution as well as on already existing equipment and characteristics of the active ingredient and chosen excipients. The most common equipment in the industry used for melt processing are the extruder, the high shear mixer, the fluid bed, the pan coater and the spray dryer, which all allow for upscaling. Depending on the desired solid dosage form, a great number of different downstream processes are addressed in the literature, such as spheronization (49,69,109,209), molding (74,95,133,183,237,238), capsule filling (87,88,91,98,100,102,139,239–246), injection moulding (247,248), freeze pelletization (121,249), pastilation (250), milling (122,196) or tableting (92,156,251). Nevertheless, it should be obvious, that manufacturing costs will rise with the addition of further process steps and, therefore, should be considered thoroughly in advance. Table IV summarizes the common advantages and disadvantages associated with the melt processing technique in the respective equipment. A comparison with the required QTPP may provide the first support for the selection of the appropriate technique and equipment.

**Table IV:** Overview Advantages and Disadvantages of Equipment Appropriate for Melting Processes

Equipment	Processing	Advantages	Disadvantages
Fluid bed	Melt coating Spray-on: sC (*in-situ*: iC) Classic: spray-on (core/shell) Melt agglomeration *In-situ* (iMA) Spray-on (sMA) Classic: *in-situ* (matrix)	• High drug load (252) • Wide range of particle sizes processable (35 μm–5 mm) (253,254) • Efficient cooling step in the same equipment • PAT-tools: Raman, NIR, particle size (255–258) • sC: ○ Adaptable for (slightly) heat sensitive drugs (short exposure to melt) (162,259) ○ relative narrow PSD (narrowing in process) (126,260) ○ sufficient taste masking with less coating prerequisite: spherical shaped, narrow PSD of core (33) • sC versus iC ○ higher coating amount applicable ○ lower risk of agglomeration • iMA versus sMA: ○ significant faster process (industry) (126, 261) ○ higher amount of insoluble excipients applicable • sMA versus iMA: more adjustable parameters to control process/product quality	• Discontinuous batch processing, minimum size dependent on machine and core density (~2.5–200 g), problem: expensive drugs • esp. sC: ○ Multitude of parameters: high level of know-how required (62,252) ○ Several requirements for core: low friability (avoids abrasion), low cohesiveness, good flowability, PSD as small as economically possible (avoids diverse coating thickness) (254,260)), regularly shaped (no needles (253)), smooth surface, bulk powder density (0.5–1.0 g/mL (253)), certain heat stability →costly or availability limited →additional costs for granulation →necessary for batch reproducibility (262) • sC and sMA: ○ limited amount of insoluble compounds in the coating (avoids nozzle clogging) (126) ○ several requirements for the excipients: *e.g.,* viscosity (~<300 mPas/80°C), melting point (~40–85°C) to prevent instabilities and nozzle clogging, narrow resolidification range (avoids agglomeration), homogenous miscibility (253) →only few suitable excipients
Pan coater	Melt coating Spray-on: sC Pour-on: pC (core/shell)	• Cheaper alternative for sC in fluid bed (41,263,264) • PAT-tools (OCT, Raman, NIR) (265–267) • sC versus pC: ○uniform film formation and high coating efficiency, better process control (264)	• Only suitable for larger particle sizes (600–5000 μm) (268) • pC: Feasibility of upscaling questionable • sC: Almost the same requirements as for sC in fluid bed
High shear mixer	Melt agglomeration Melt-in: mMA Spray-on: sMA (pour-on: pMA) Classic: mMA (matrix) Hot fusion (HF) for pre-formulation studies and/or further downstream processing (solid dispersion)	• Multifunctional mixing device, may avoid investment for specialized equipment • PAT-tools (Raman, NIR) (269,270) • HF/ mMA: Convective mixing mechanism: applicability to a wide variety of drug formulations (*e.g.,* fine, cohesive material, broader PSD, binders with higher viscosity *etc.*) (271,272) • mMA: Simple, less process parameters (273,274)) • sMA versus mMA: More adjustable parameters for finer process control	• esp. mMA/HF: ○ Inappropriate for thermosensitive drugs ○ Chemical reactions are accelerated • mMA/HF: ○ Time-consuming cooling step necessary (further cooling equipment might be required) • esp. mMA: Denser product compared to fluidized bed, reduced compressibility and dissolution rate (275–278)
Extruder	Melt extrusion (Hot fusion) and shaping through die (Solid dispersion) Downstream auxiliary equipment for cooling, further shaping (*e.g.,* pellets) and particle size reduction (279)	• Adaptable for heat sensitive drugs: application of low recrystallizing excipients (43), reduction of residence time, or addition of antioxidants (279) • High content uniformity (280) • Various downstream processing methods and dosage forms are available (280,281) • Fast, continuous manufacturing feasible (comparably easy scale up) (282,283) • Wide variety of suitable formulation strategies (*e.g.,* bioavailability enhancement (284), controlled release, taste masking (281) *etc.*)	• High level of know how required, high modularity of process • Formulation development time- and material-consuming (282) • Additional expensive downstream equipment required • Storage stability might be an issue (*e.g.,* recrystallization of amorphous drug) (284)
Spray dryer	Spray congealing Hot fusion before spraying into cooling chamber Depending on nozzle type: Microsphere (Matrix) Microcapsule (core/shell)	• Fast cooling step • Highly spherical particles with smooth surface (285,286) • Different atomization methods applicable: wide particle size range (~10–6 mm) (286) and high through put achievable (287) • Comparably easy upscaling (285) • PAT-tools: laser diffraction, NIR (288,289)	• Limited drug load (solid API: 30%, liquid API: 50%) (285) • Unsuitable for heat sensitive drugs, degradation in melt (285) • Original particle size must be significantly smaller than desired product size. Additional milling step might be necessary (286) • Additional melt-mixing equipment required • Resolidification of larger particles requires longer cooling towers or special cooling methods (286)

The following chapter will focus on the developed formulations and associated characteristics of the final product, on the basis of the categories melt extrusion, melt coating and melt agglomeration. At this point it is worthy of note that the terms for the melting techniques are not used consistently in the literature, especially when the topics melt coating and melt granulation are concerned (51). The fundamental differences between these techniques lie in the distribution of the API and the size of its contact surface with the lipid excipient. Melt extrusion provides a homogeneous solid dispersion after processing, while the distribution is less homogenous and the contact surface of the API and the lipid is smaller when melt agglomeration by high shear mixing and in particular melt coating are used. Nevertheless, the delimitation between these terms is complicated. For instance, several downstream processes of melt extrusion allow for the formation of pellets and granules, and with melt coating techniques a drug / lipid dispersion may be sprayed on nonpareils creating a solid dispersion layering.

### Melt Extrusion

The classic equipment for the melt extrusion process is the extruder, which consists of a single- or twin-screw system. In both systems the screw is positioned in the center of a heatable stationary barrel. Three zones within the extruder are named after their individual function.

1. Feed zone: maximum pitch between barrel and screw flight
  The feed material enters this zone through a gravimetric or volumetric hopper, is mixed under low pressure and transported by the screw rotation to the compression zone.
2. Compression zone: continuous reduction of the pitch along the screw
  The steady pressure increase leads to compression, particle size reduction and more effective shearing and mixing. The heated barrel wall and the frictional heat are used to melt or at least soften the material. Additional equipment such as conveying and kneading elements may be used for the mixing, homogenization, venting and kneading process.
3. Metering zone: constant minimum pitch
  The continuous high pressure supports a homogenous and steady material flow to and through the die.

After melt extrusion the product is cooled and shaped downstream in further processing units, such as hot strand cutters, calendaring, chill rolls, or spheronizers/marumerizers (128,280,281,290 294).

Melt Extrusion is widely used for two main purposes, namely enhancing the bioavailability of poorly soluble drugs and the development of formulations with modified release profiles (294–296). Jannin *et al.* (9) and Keen *et al.* (247) reviewed a number of formulations showing an improved drug release. The preferred lipid excipient was Gelucire 44/14, which was extruded in a blend with 17 β-estradiol and PVP, PVA, or PEG 6000 (92). Mehuys *et al.* extruded Propranolol with HPMC and Gelucire 44/14, which was molded as core material in an ethyl cellulose pipe. The ethyl cellulose pipe offers protection against hydrodynamic and mechanical stress and provides a sustained, zero-order, erosion-controlled drug release and a better bioavailability in dogs compared to a commercial product (237,297,298). The application of extruded wax matrices for the retardation of drug release started in the early 1990s (299,300). Sato *et al.* and Miyagawa *et al.* conducted a study to analyze the influence of dissolution rate-controlling agents on extruded carnauba wax matrices with diclofenac as model drug. Their investigations highlighted the importance of a proper pre-selection of the dissolution modifiers and their physiochemical properties (301,302). Liu *et al.* prepared tablets from sustained-release extrudates of phenylpropanolamine, wax and different types of fillers and showed the effect on the dissolution rate caused by the type of filler in the matrix (303). This matches the investigations of De Brabander *et al.*, who described a different impact on dissolution with different starch derivatives in extrudates compressed to mini tablets (304). Quintavalle *et al.* developed a sustained co-extrudate with an inner hydrophilic core (PEG 6000) and an outer lipophilic coat both containing theophylline as drug. Its release was tailored through a suitable selection of parameters. In addition a very small *in vivo* bioavailability study of four healthy volunteers confirmed the desired sustained release (305). Roblegg *et al.* introduced vegetable calcium stearate for preparing sustained release pellets with a 20% paracetamol drug load. Although the addition of dissolution enhancers like glyceryl monostearate and tributyl citrate was able to reduce the process temperatures, the temperature of 70°C is still inappropriate for thermolabile substances (128). Vithani *et al.* successfully applied Compritol® 888 ATO as an excipient for the extrusion of the model drug diclofenac sodium and subsequent direct compression of sustained release tablets (306). Several investigations from the working groups of Breitkreutz and Kleinebudde *et al.* delivered insight into the applicability of different lipid excipients and the polymorphic behavior after melt extrusion and storage (43,131,142,159,161,163). Their storage stability studies revealed a superior stability of homogenous lipids (*e.g.,* Dynasan 114) and emphasized the requirement for understanding and avoiding polymorphic transformation during storage (43,142). With Witocan 42/44 melt extrusion at room temperatures (“solvent-free cold extrusion”) and a stable release profile over storage was feasible (42,43). Another approach for reduction of the process temperature and extending the release was adopted by Schulze and Winter for a protein co-lyophilisate of INF α with hydroxypropyl-β-cyclodextrin in a lipid implant. The selective melting of a low melting lipid excipient in a blend with a high melting lipid provided an extrudable mass at moderate temperatures, an unchanged protein structure and avoided polymorphic changes of the high melting lipid due to recrystallization (207). In a second study with lysozyme Sax and Winter chose different low melting lipids in the combination with Dynasan 118 and different portions of pore forming agent to analyze the influence on the release kinetics of protein molecules from the lipid implant (45). The partial melting of the low melting fraction at incubation temperatures of 37°C built a protein reservoir and an additional non-aqueous diffusion pathway besides the conventional way through the buffer filled interconnected pore-network created by the dissolution of hydrophilic excipients (45,165). Recently, Oliveira *et al.* evaluated the extrudability of formulations, comprising Gelucire 33/01 and two further lipid excipients such as Dynasan 114, Precirol ATO 5, Witocan 42/44 or Compritol 888 ATO at room temperature and revealed a complex influence on porosity, thermal behavior, mechanical properties and dissolution rate depending on the chosen composition (307). It can be concluded that if lipid excipients are used, not only the dissolution rate but also mechanical properties and porosity should be monitored during storage. A list of formulations developed *via* extrusion and their characteristics are summarized in Table V.

**Table V:** Overview: Selection of Formulations and Characteristics

API	Lipids	Application/product characteristics
Sodium benzoate (42)	Stearic acid, Precirol ATO 5, Witocan 42/44	Taste masking Witocan 42/44 with superior binding capacity even at room temperature (cold extrusion) Solvent-based coating with Eudragit E required for taste masking
Theophylline (142)	Precirol ATO 5, Dynasan 114	Controlled release Dynasan 114: porosity dependent on extrusion temperature (“blooming”) Precirol ATO5: alteration in dissolution rate during storage (“ageing”)
Enrofloxacin (196)	Compritol 888 ATO/Aerosil 200	Taste masking Original die diameter: impact on dissolution rate of very slightly soluble drugs Extrusion temperature: no impact on dissolution rate
Sodium benzoate (43)	Witocan 42/44/Dynasan 114, Precirol ATO 5, Compritol 888 ATO	Immediate release (taste masking) Cold extrusion feasible for binary/ternary mixtures of Witocan 42/44 and other lipids Precirol ATO5: ageing during storage at elevated temperatures led to delay in dissolution rate
Theophylline diprophylline (44)	Witocan 42/44, Precirol ATO 5, Dynasan 114	Controlled release Faster drug release from extrudates with Dynasan 114 in comparison to Precirol ATO 5 Impact factors on dissolution rate: particle size and drug load
Theophylline (159)	Dynasan 112, Dynasan 116, Dynasan 118	Controlled release Diffusion controlled and chain-length dependent dissolution rate; extrusion temperatures below melting point of instable α-form may induce storage instabilities
Theophylline (131)	Dynasan 118/Imwitor 491	Controlled release Diffusion controlled dissolution, Imwitor 491 stabilizes the α-form and leads to storage instability due to transformation into the stable β-form and the formation of water repellent fractal structures
Theophylline (161)	Dynasan 116/polyethylene glycol 10000	Controlled release Dissolution rate dependent on amount of additional polyethylene glycol 10000 amount; process temperatures above α-form avoid polymorphic transformation during storage
Trospium chloride (163)	Dynasan 118	Controlled release Extrudates show a fast initial and slower release over days. Tempered mini-molds have a negligibly initial drug release, an appropriate retardation and exhibit the stable polymorph.

All in all, lipid-based excipients usually show a lower melt viscosity and melting point compared to polymeric excipients typically used in hot melt extrusion (*e.g.,* hydroxypropyl methylcellulose, cellulose acetate, polymethacrylate) (294). Hence, with lipid excipients the addition of plasticizers (*e.g.,* PEGs, triacetin, citric acid, GMS *etc.*) to decrease the glass transition point and melt viscosity is dispensable. This leads to a reduction of formulation complexity and allows for lower process temperatures (294,308,309). Thus, lipid excipients are able to extend the portfolio of suitable APIs for hot melt extrusion by also including heat sensitive candidates such as proteins (207). Another important issue in the recent years was the adjustment of the dissolution rate. Most of the published studies focus on sustained release formulations (128,142,303,306,310) but only a few on the application for taste masking by maintaining the immediate release profile (42,43,233,235) and enhancement of the solubility of poorly water-soluble drugs (92). The enhancement of the release profile was usually performed by adding rather high amounts of additives such as polyethylene glycol (161), or certain amounts of polymers such as PVP (92). However, the literature seems to lack in successful solvent-free melt extrusion approaches with lipid-based excipients alone, although hot melt extrusion with polymers is one of the most popular methods to enhance solubility (281). The reasons for the reluctant development of these kinds of formulations are multifactorial. The choice of lipid-based excipients for oral application suitable for this purpose is rather limited. The idea of including polyethylene groups is perhaps sufficient to increase the HLB and solubility, but leads to low melting temperatures (Gelucire 44/14—HLB 14, melting point 44°C) impeding melt processing (*e.g.,* in spheronization step) (92) and deteriorating formulation stability. Additionally, changes in polymorphism may occur and alter dissolution rates during storage (*e.g.,* Gelucire 50/13—HLB 13, melting point 50°C) (311). Also other excipients such as sucrose esters, which would be stable up to 140°C (312) and are available with a promising high HLB of 16, showed polymorphic transformation during storage after melting (173). Polyglycerol esters crystallize in hexagonal subcells, exhibit no polymorphic transformation, are thermally stable below 100°C (312) and available in a HLB range from 6 to 11 (313). However, only one study published in 1993 could be found where these excipients were successfully applied in a spray chilling process (314).

### Melt Coating

The first publication mentioning the term “melt coating” or more precisely “hot melt coating” was an US patent published in the year 1942 (315). A solvent-free coating technology was described to coat sheet material (*e.g.,* paper, foil, metal) with molten thermoplastic resins, which can be used for the manufacturing of water- and grease-proof primary packaging of food (315,316). In the following decades the coating with molten lipid-based excipients became a standard procedure in the food industry (317,318). The literature discusses a wide range of food products coated by recrystallization of lipid-based molten excipients. For example: dip-coating of frozen meat (“larding”) (317,319,320), spray- or dip-coating as well as enrobing of ice cream with molten chocolate (321), spray chilling/congealing or fluidized bed coating of supplements (*e.g.,* vitamin C) (322), food additives (*e.g.,* aspartame) (323), flavors (324), or even heat sensitive probiotic bacteria (47,259), to name just a few. The advantages of this coating method have not gone unnoticed by the pharmaceutical industry. In the 1960s spray chilling was used for the first time by Merck & Co Inc. to achieve taste masking for iron particles (325) as well as for water-soluble vitamins (326). In 1968 Richardson Merrell Inc. applied sprays of molten resin mixtures on a tumbling bed of tablets in a pan coater (327,328), which marked the birth of the so called pan spray coating procedure. This technique was also transferred to a fluid bed coater, which was equipped with an additional heating system for the atomization air by Glatt Air Techniques Inc. in the early 1990s (62, 329). This was the start of further research and development in this field, which has been reviewed in several articles (13,253,263,264,330,331). The fluidized bed coater, the pan coater and also the spouted bed are common machines used for hot melt coating (264,332–334). In a coating pan the molten material is either sprayed by a nozzle (pan spray (63,327,328)) or poured onto the rolling product (pan pour coating (113,167)). The same can be performed in a fluid bed chamber by fluidizing and melting the lipid excipient together with the product (*in-situ* hot melt coating/ solid dispersion fluid bed hot melt coating (123,147,335)) or by spraying (hot melt spray coating (62,221)). Classically, the drug is preheated and fluidized in the fluid bed coater, but heat stable drugs can also be homogenously dispersed in the molten coating material and sprayed on fluidized nonpareils seeds (drug lipid dispersion hot melt coating (64–66)). This makes a time-consuming process step to obtain a core material with appropriate particle characteristics (*e.g.,* size distribution, flowability, friability *etc.*) superfluous. Nevertheless, this method is only suitable for a small drug load as the solid amount in the spray dispersion can clog the nozzle during spraying. Drug-lipid interactions have to be evaluated in advance, as the drug may be dissolvable in the lipid matrix and can be present in the amorphous state after resolidification (65) posing a potential risk of formulation instability due to recrystallization during storage. Further, the particle size of the drug can have a significant impact on drug release (65) and therefore has to be observed during formulation development. However, pouring techniques and *in-situ* hot melt coating are used more often in the context of research than in large-scale manufacturing. The use of pouring techniques in pan coating requires a high degree of attention and care, as poor execution may cause twinning as well as inferior coating homogeneity and efficiency (52). *In-situ* hot melt coating can be used without any additional hot-melt equipment (*e.g.,* nozzle, heating system *etc.*), which may be advantageous for faster pre-studies. Nevertheless, the applicability in production scale is questionable, as core materials smaller than 420 μm are prone to agglomerate, porosity and particle size distribution affect batch-to-batch reproducibility and the lipid amount successfully applicable without agglomeration is rather low (<5%) and fails to reach the target specification (335). Hot melt spray coating requires more experience for the parameter selection, additional heating equipment, excipients with appropriate physicochemical characteristics (*e.g.,* melting point, viscosity *etc.*) for the spraying process, and more time for the spraying step. Nevertheless, it is preferred over *in-situ* melting as it offers the option of fine adjustment of the product quality due to a higher number of process parameters, a lower risk of agglomeration, minor thermal stress of the API due to significant shorter exposure times to the molten coating, and easier up-scaling. Table VI provides a selection of formulations produced by spray coating and their intended purpose found in the literature.

**Table VI:** Overview: Selection of Formulations and Characteristics

API	Coating agent	Characteristics/application
Acetaminophen	Carnauba wax/sorbitan monostearate Hydrogenated vegetable oils/sodium stearyl lactate or sorbitan monostearate or glyceryl monostearate (46)	Taste masking, immediate release, storage stability achieved with carnauba wax, formulation with hydrogenated vegetable oils with inferior storage stability
	Compritol 888 ATO (152)	Controlled release (Higuchi model)
	Precirol ATO 5, stearic acid Compritol 888 ATO Several combinations with surfactants and/or release enhancer were tested: CaCO_3_, PEG 3000, PEG 4000, Amberlite IRP, Tween 20, Cremophor EL, Cremophor A6, Gelucire 50/13, Kollidon CL-M, Kollidon CL, Carbopol 971P NF, Carmellose Sodium, KHCO_3_, Lactose, Blanose (336)	Immediate release Taste masking assumed, only study of release profile, no study for taste masking or formulation stability included
N-acetylcysteine (162)	Tripalmitin/Polysorbate 65	Immediate release, taste masking (volunteer panel), stability achieved, acceleration of α → β transition due to emulsifier
Antibiotics (40)	Carnauba wax/carbomer, xanthan gum, L-HPC	Immediate release, taste masking (electronic tongue), storage stability achieved
Bromhexin HCl (33)	Bees wax/cetyl alcohol	Taste masking (human volunteers) Stability not approved
Chloroquine (145)	Compritol 888 ATO	Controlled release
Diclofenac sodium (111)	Stearic acid, palmitic acid	Enteric coating
Diltiazem HCl (337)	Glyceryl monostearate/bees wax/ white wax/ stearyl alcohol	Immediate release, taste masking was assumed (drug release after 1 min) Stability not approved
Herbal extract (115)	Stearic acid/ PEG 6000	Immediate release, moisture sorption control, stability not approved
Ibuprofen	Precirol ATO 5 (27)	Immediate release, taste masking and stability not approved
	Compritol 888 ATO (146)	Controlled release Maturing reduced and stabilized dissolution rate
Metoprolol tartrate (34)	Bees wax/ethyl cellulose (34)	Sustained release, stable during storage
Phenylpropanolamine (146)	Compritol 888 ATO	Controlled release Maturing reduced and stabilized dissolution rate
Theophylline	Hydrogenated castor oil/HPMC, sodium laurel sulphate (52)	Controlled release Hydrophilic pore formers increased the release, no stability study conducted
	Compritol 888 ATO (143,144,146)	Controlled release

Hot melt coating by direct blending is another method more recently mentioned in the literature (338,339). Drug granules or nonpareils are stirred in the molten coating or coating drug dispersion until homogeneity is reached and then congealed by cooling down to room temperature under continuous, vigorous stirring (37,339). A zero-order release was obtained by double coating of extruded acetaminophen pellets with carnauba wax and HPMC in the inner and Syncrowax HR-C (glyceryl tribehenate) in the outer coating. HPMC and EC were described as useful swelling and eroding agents, which are able to decrease initial burst release by hindering drug diffusion (338). Further, sugar spheres with a coating comprising a waxy excipient with a low melting point and higher polarity (*e.g.,* cetostearyl alcohol, Polawax, and 1-Monostearin) and a low drug load of acetaminophen exhibited an immediate release profile. At the first glance, the processing seems very convenient and easily controllable for particles with an appropriate size (250–595 μm) without the requirement of any sophisticated equipment in laboratory scale (37), but if and how this method is suitable for up-scaling has yet to be evaluated. It will also be of further interest, if taste masking can be attained by this approach, how the dissolution profile can be tailored by different pellet and coating compositions (*e.g.,* other drugs, higher drug load, multi-layers) and most importantly if the obtained formulations are stable during storage.

As already stated in the subchapter on hot melt extrusion, controlled release was the main application purpose for hot melt coating with lipid-based excipients. Rosiaux *et al.* gave a comprehensive insight into the literature on drug release mechanisms of sustained release lipid matrices (26). Immediate release was achieved with a homogenous mixture of a lipid excipient with a rather low HLB (HLB ≤ 5), a higher melting point (≥60°C), a small recrystallization range (≤35°C) and a hydrophilic polymer or emulsifier with sufficient miscibility in the melt (*e.g.,* hydroxyethyl cellulose, hydroxypropyl cellulose, carbomer, PEG-derivatives, E-400–E-499 *etc.*) (40,336). The emulsifiers and disintegrants were mainly selected on the basis of their HLB and hydrophilic behavior, but only one study is known in which the emulsifier was used as a polymorphic modifier to gain the stable β-phase during and directly after the process (162). Although studies on storage stability are the most crucial point while working with lipid based formulations, in particular if complex coating compositions are used, only very few studies have taken this point into account (40,46,162). The same holds true for the evaluation of taste masking, which often is a further target of this kind of formulation. Sufficient taste masking is often assumed on the basis of the release profile in the first minute of dissolution, but only rather seldom volunteers or more sophisticated analytical tools are used for the evaluation (40).

### Melt Agglomeration

Melt agglomeration includes melt granulation and melt pelletization, which provides highly spherical agglomerates with a narrow size distribution and a particle size from 0.5 to 2 mm. However, both terms cannot be clearly distinguished and are not used consistently in the published literature (340,341). Apart from using a melt extruder equipped with the necessary accessories, the formation of granules and pellets can be performed in a fluid bed (84,124,342,343) or high shear mixer (55,75,81,99,130,148,169,344). Schaefer *et al.* gave a deep insight into the important process parameters especially for the high shear mixer and described the following phases and mechanisms during agglomeration (125,345–350).

1. Nucleation phase (345,346,351,352):
  In the first step the solid lipid binder is either filled into the equipment together with the starting material consisting of excipients (*e.g.,* filler, disintegrants *etc.*) and/or drug or molten externally with or without the drug. In case the binder is molten during mixing with the remaining compounds the method is described as “melt-in” (high shear mixer) or also “*in-situ*” (fluid bed) agglomeration. If the binder is added in the molten form it can either be sprayed with a nozzle onto the preheated material in the “spray on”-method or steadily pumped onto the preheated material in the “pour-on” or “pump-on” method. Two different nucleation mechanisms after wetting of the material have been postulated. One is the distribution mechanism where the molten binder spreads over the surface of the starting material and primary nuclei are formed by coalescence. The other is the immersion mechanism, which is predominant if the molten binder droplets are larger than the starting material particles and the solid particles immerse into the molten droplet surface to form the nuclei. The droplet size can be affected by the shear rate (melt-in, pour-on), the initial particle size of the lipid binder (melt-in), the spray rate and pressure (spray-on), as well as by the binder viscosity at the applied temperature. The nucleation takes place as long as nuclei interact with initial particles. This leads to a depletion of fines and finally to an increased wetting and starting of growth by coalescence between nuclei.
2. Growth phase (345,346,351,352):
  The growth phase presents an equilibrium between consolidation and growth until a critical size is reached and attrition and breakage into smaller particles takes place. The critical size depends on several parameters such as the physicochemical properties and amount of the binder (*e.g.,* particle size, viscosity, deformability, temperature *etc.*), characteristics of the starting material (particle size, shape, density *etc.*) and kinetic energy applied (impeller speed and frictional heat, air temperature) (346,351,352). Parameters such as the particle size and shape, the binder viscosity and a certain solid/lipid ratio also have an effect on the granule strength and whether agglomerates are formed and densified by coalescence or if breakage to fragments and layering on the existing agglomerates is present. In case the binder viscosity and lipid/solid ratio are well-chosen, densification and a steady growth will take place and depending on the binder amount and the applied shear force, particle size distribution narrows, sphericity increases and porosity decreases (melt pelletization).
3. Cooling phase (81,352)
  The cooling phase can be performed by cooling the particles in the equipment, which is much faster in the fluid bed than in the high shear mixer due to the better heat transfer, or by rapid cooling by pouring the material directly into liquid nitrogen (“flash-cooling”) or by simply spreading the material out in thin layers on trays (high shear mixer). In case of polymorphic material or material that can vary in the degree of its crystallinity (*e.g.,* PEG 3000), the cooling rate can play an essential role in controlling formulation stability and drug release (81). Table VII lists a selection of formulations prepared by melt agglomeration in a high shear mixer and fluid bed.

**Table VII:** Selection of Formulations Produced by Melt Agglomeration Techniques

High shear mixer		
API	Excipients	Application
Acetaminophen	Glycerol monostearate/ aminoalkyl methacrylate copolymer E (353)	pH-dependent drug release (appropriate for taste masking)
	PEG-6-stearate (169)	Rapidly disintegrating tablets and increased physical resistance Waxy excipient with melting temperature lower than in the body (33–37°C) and high HLB of 9
	Stearic acid (187,188,274)	Sustained release Increased bioavailability *in vivo*
Diazepam (81)	Gelucire 50/13	Dissolution enhancement Applied drug load: 30–40% Similar dissolution for pump-on and melt-in method
Dipeptidylpeptidase IV Inhibitor (50)	Hydrogenated castor oil	Moisture protection, maintained immediate release
Lansoprazole (71,75)	Gelucire 44/14, Gelucire 50/13	Dissolution enhancement Box–Behnken design inputs: binder concentration, batch size, mixing time, impeller speed
Griseofulvin (99)	Gelucire 44/14	Dissolution enhancement Applied drug load: 2.5–5% 2^4^ factorial design Input: drug load, binder, filler and HPMC
Phenylephrine HCl (138)	Precirol ATO 5/Compritol 888 ATO	Sustained release Instable in accelerated storage conditions
Riboflavin (148)	Precirol ATO 5/Compritol 888 ATO	Floating formulation Increased urinary excretion *in-vivo* especially after feeding.
Theophylline (149)	Precirol ATO 5/Compritol 888 ATO	Floating formulation Gas generation agent: sodium bicarbonate Drug load >40%
Fluid bed		
API	excipients	Application
acetaminophen	Precirol ATO 5 (198)	Taste masking Volunteer study *In-situ* method 2^3^ full factorial design Inputs: binder particle size, content, granulation time, air flow rate Highly spherical particles
	Gelucire 50/13 (354, 355)	Immediate release Applicable granules for tableting *In-situ*, spray-on 2^3^ full factorial design Inputs: binder content, spray rate, spray pressure Box-Behnken design, multilayer perceptron neural network Binder size controls granule size and shape
Ibuprofen (356)	Precirol ATO 5/Gelucire 54/02	Controlled release and lubrication *in-situ* Appropriate granules for tableting
Lu–X (84)	Glycerol monolaurate, Gelucire 50/13	Dissolution enhancement Melt-in and spray-on Distribution or immersion depending on the binder, difference in dissolution Stable during storage at 25°C/3 months

Several designs have been applied to understand the granulation mechanisms and the effect of different process parameters on product quality (71,99,118,198,274,357). Comparison studies using in-line particle size measurement tools (focused beam reflectance measurement, spatial filter velocimetry) for the endpoint determination between the binder application methods (melt-in/*in-situ*, spray-on) revealed that the results were comparable in respect of particle size distribution and flowability. The dissolution profiles of the granules processed in a fluid bed and in a high shear mixer were comparable, provided the drug was applied in the same close contact to the lipid excipients (126,277). Further studies revealed that both fluid bed methods, spray-on as well as *in-situ*, were able to obtain smooth and spherical granules with a comparably fast dissolution rate for granules with the same size, while the particle size distribution seems to be positively affected by smaller binder particles or droplets in both methods (354). Studies revealed that melt agglomeration in a fluid bed can be successfully applied to improve the lubricant performance, flowability, and compressibility of the granules in a subsequent tableting step (358,359). This is caused by a homogenous repartition and due to the significantly lower shear force in a fluid bed than in a high shear mixer that leads to a reduction of particle densification and to an increased elastic deformation and better compressible granules (124). Moreover, the fluid bed offers a more efficient cooling process without additional densification, which is an advantage for up-scaling and enables the addition of higher binder contents (343). Further, Kukec *et al.* were able to successfully scale an *in-situ* laboratory method up to pilot scale by the aid of a three-factor, five-level circumscribed central composite design, whereby the resulting dissolution rate was affected mainly by the binder content (261). Hence, *in-situ* melt fluid bed granulation is a viable and fast method to obtain granules from moisture sensitive drugs.

### Other Methods

#### Spray Congealing (Synonyms: Spray Chilling, Spray Cooling)

Although spray congealing is often listed as a melt agglomeration technique (340,352,360), it can be seen as a mix of all three aforementioned methods, as the products are perfectly spherical microspheres or microcapsules that can contain the drug embedded as a solid dispersion in the lipid matrix. The nozzle type and fluid delivery determines whether a matrix (“microsphere”) or a more core/shell-like structure (“microcapsules”) of the drug embedment is generated. Prilling is a special form of spray-congealing that delivers a product with an increased particle size of 500 to 2000 μm (“prills”) (120,157,361). For spray congealing a stable and homogenous mixed melt dispersion of the drug (and further excipients if necessary) is required. The chosen technique (*e.g.,* ultrasound homogenizer, high shear mixer) for the preparation of the dispersion may have a significant impact on the rheological behavior, particle size and crystallinity and should be therefore controlled during product development and processing (76). This mixture is subsequently processed in a choice of atomization units, which are distinguished by their atomization mechanism, liquid channeling, throughput and product properties (*e.g.,* particle size and distribution, structure *etc.*) (285,286). Examples mentioned in the literature are the spinning disk (127,132,362,363), vibrating nozzle (157,361), pneumatic nozzle (101,120,364,365), ultrasonic devices (72, 77,78,366–368), or dual-fluid nozzle (73,76). After atomization the particles are solidified by falling in a large prilling tower, a chamber of a spray dryer flushed with cold air, liquid nitrogen or cooled by a carbon dioxide ice bath (285,364). This step is critical especially if greater particles like prills have to be solidified, which may lead to deformation or sintered lipid blocks in the product container due to an inappropriate time of flight and an incomplete recrystallization. Table VIII gives an overview of some formulation characteristics obtained with spray congealing.

**Table VIII:** Selection of Formulations Produced by Spray Congealing

API	Lipid excipients	Characteristics
Acetaminophen (127)	Glycerol monostearate	pH-dependent release (taste masking) Box–Behnken design Inputs: drug load, Eudragit E amount Mean size: 400 μm Drug: 10–30%
Glimepiride (73)	Gelucire 50/13	Dissolution enhancement Morphological changes (“blooming”) during storage (30°C/1 month) d_50_: 58–278 μm Drug: 1.7% (*w/w*)
Meloxicam (101)	Gelucire 44/14	Dissolution enhancement Drug load: ~ 10%
Mesalazine (36)	Carnauba wax, stearic acid	pH-dependent release Two step congealing process 1. carnauba wax/drug reservoir 2. stearic acid enteric coating Drug load: ~ 18%
Metoprolol tartrate (120)	Stearic acid, behenic acid	Sustained pH-dependent release Prilling: 1.8–2.5 mm Drug load: 10–40% Polymorphism of fatty acids not affected Drug/lipid interaction, amorphous fraction recrystallized during storage at accelerated conditions *In vivo* results comparable with commercial formulation

An important point to consider while working with the spray congealing technique is the polymorphic state of the obtained microspheres. As rapid cooling is applied, thermodynamically unstable forms crystallize during the resolidification step. Even microspheres with carnauba wax, which is often stated to be a non-polymorphic material (40,46,221), showed thermal transition events in a microcalorimetric system during storage. However, the effects on parameters such as the dissolution rate were not evaluated (362). A clear polymorphic change was detected with tripalmitin-insulin microspheres, which showed the instable α-form after resolidification and transformed into the stable β-form within 28 days of storage (365). Yajima *et al.* discovered that the transformation of the α-form of the freshly spray congealed glycerol monostearate Eudragit E microspheres to more stable polymorphic forms had a significant impact on the drug release in a mini-column method used for testing taste masking efficiency (202). Li *et al.* observed that the drug Bupivacaine as well as tristearin crystallize in their unstable form during spray congealing and the transformation into their stable forms during heat-treatment has a significant impact on the fluidity and gelation behavior in an aqueous medium (363). Although Gelucire 50/13 was able to enhance the dissolution rate of Piroxicam in spray congealed microspheres significantly, the morphological structure changed from a smooth surface to a flake-like structure (“blooming”) and the dissolution rate increased during storage (80). Additionally, even the drug showed a remarkable impact on the polymorphic transformation behavior, for instance, paracetamol was able to stabilize the low melting lipid fraction while caffeine was associated with the transformation to more stable phases (74). Moreover, different polymorphic forms can exhibit a different potential to incorporate drugs in a molecular dispersed form, and therefore changes in lipid polymorphism during storage can lead to drug precipitation and changes in dissolution rate (214). But also the crystallinity and polymorphic state of the drug are important, as melting techniques are able to generate an amorphous state of the drug or reduce crystallinity or have an effect on the recrystallized polymorph of the drug (120,364,369). Hence, studies on polymorphism and drug/lipid interactions are crucial for formulation development.

#### Freeze Pelletization

Freeze pelletization is a variant of the spray congealing process. The molten dispersion is dropped into a column with a cooling liquid with a needle or nozzle (121,249,370). The liquid offers a better heat transfer than the air and the resulting pellets are described as nonporous, spherically shaped and exhibiting a narrow size distribution (370). Nevertheless, the selection of a suitable cooling fluid seems to be a great challenge, as is has to be non-toxic, inert, immiscible and has to have an appropriate viscosity (370).

#### Pastillation

Pastillation is a method to facilitate the handling with dusty hazardous powders by transforming them into pastilles common in the petro- and agrochemical industry (250). In this process the molten drug/lipid dispersion is dropped with a needle on a cold surface. An important parameter is the contact angle of the solidified drop to the surface, which affects the flowability of the pastilles and can be adjusted by the needle height and geometry as well as with the temperature of the surface (250,371,372). As the up-scaling to continuous manufacturing seems to be rather easy and different lipid-materials should be applicable, this method seems to be promising for the pharmaceutical industry, provided recrystallization behavior and Reynolds number are appropriate (371).

#### Fusion (Synonyms: Hot Fusion, Melt Fusion, Melt-Mixing) and Melt-Solidification

The term “hot fusion” refers to the preparation of a solid dispersion of a drug in a matrix and therefore can be understood as a kind of melt extrusion but with the use of less sophisticated equipment on a laboratory scale and has often been applied in pre-formulation studies. Hot fusion is performed by melting the lipid excipient and mixing the drug homogenously by using a magnetic stirrer, a high shear mixer or rotor-stator homogenization to generate a solid dispersion or even a solid solution (29,32,85,153,369). The molten mixture can be molded into tablets (68,373–375), filled in capsules (102,139), poured into a cold water bath and stirred to obtain beads (110,376,377), screened through a sieve for generating granules (32,378), spheronized to pellets (49), or ground and sieved (48,114,136,153,172). The sieved material may be used for direct compression to tablets (32,48,114,136,153,158,169,172,379). The fusion of highly soluble drugs with hydrophobic matrices (*e.g.,* Compritol 888 ATO, glyceryl monostearate, stearic acid, Precirol ATO 5, hydrogenated castor oil *etc.*) retards the drug release (29,48,114,136,251) and tableting of this solid dispersion has a significantly higher efficiency to sustain release than the direct compression of the physical mixture (29,136,153,210,379). The dissolution of poorly water soluble drugs may be significantly enhanced in a solid dispersion with hydrophilic lipid excipients, such as Gelucire 50/13 (85), Gelucire 44/14 (89,93) compared to the physical mixture of the same (85,93). The addition of an effervescent formulation to the tablets can also help overcome the slower release of tablets compared to an according multiparticulate system (89).

#### Sintering of Tablets

Thermal treatment (“sintering”) of tablets received by direct compression of the physical mixtures had a significant retarding effect on the dissolution rate due to a redistribution of the wax and an increased matrix tortuosity (158,380,381). However, with hydrogenated cotton seed oil the opposite effect has been seen, which was assumed to be a consequence of wax migration (379). As the study lacks solid-state analysis data a change in polymorphic and morphological properties cannot be excluded (379).

## Conclusion

Solvent-free melting techniques are well-known in the literature and very promising for the pharmaceutical industry. The achievable formulation properties serve a wide-area, ranging from modified release by allowing different dissolution kinetics, but also bioavailability enhancement (*e.g.,* gastroretentive formulation), taste masking, up to moisture protection and improvement of swallowability. A recent trend is the design of multiparticulate drug delivery systems, which strike a good and balanced compromise between taste masking and fast release. These systems play a key role in the development of population driven patient-centric strategies, improving the adherence to medication for swallowing difficulties in pediatrics and geriatrics (382). An ongoing challenge in working with lipid materials is the proper handling of stability issues such as polymorphic and morphological changes. Meeting this challenge is worth the effort: the materials required are often cheaper than the corresponding polymers and are mostly processable in standard industrial equipment such as the high shear mixer or fluid bed coater. This facilitates the decision for feasibility studies utilizing a solvent-free melting process in formulation development. Therefore, solvent-free melting processes offer an effective, simple, safe and eco-friendly way of manufacturing in pharmaceutical and food industries.

## ABBREVIATIONS

API: Active pharmaceutical ingredient
BET: Brunauer–Emmett–Teller theory
Caco: Colon adenocarcinoma cells
DSC: Differential scanning calorimetry
DVS: Dynamic vapor sorption
EDX: Energy dispersive x-ray microanalysis
FDA: Food and drug administration
FT-IR: Fourier transform infrared spectroscopy
GRAS: Generally recognized as safe
HLB: Hydrophilic lipophilic balance
HSM: Hot stage polarization microscopy
IIG: Inactive ingredient guide
INF: Interferon
NIR: Near infrared spectroscopy
PAT: Process analytical technology
PEG: Polyethylene glycol
PgP: P-glycoprotein
SEM: Scanning electron microscope
TGA: Thermogravimetric analysis
XPS: X-ray photoelectron spectroscopy
XRD: X-ray diffraction
